# Development of a Freeze-Dried Fungal Wettable Powder Preparation Able to Biodegrade Chlorpyrifos on Vegetables

**DOI:** 10.1371/journal.pone.0103558

**Published:** 2014-07-25

**Authors:** Jie Liu, Yue He, Shaohua Chen, Ying Xiao, Meiying Hu, Guohua Zhong

**Affiliations:** 1 Laboratory of Insect Toxicology, and Key Laboratory of Pesticide and Chemical Biology, Ministry of Education, South China Agricultural University, Guangzhou, P.R. China; 2 Guangdong Zhuhai Supervision Testing Institute of Quality and Metrology, Zhuhai, P.R. China; 3 Guangdong Province Key Laboratory of Microbial Signals and Disease Control, South China Agricultural University, Guangzhou, P.R. China; Federal University of Viçosa, Brazil

## Abstract

Continuous use of the pesticide chlorpyrifos has resulted in harmful contaminations in environment and species. Based on a chlorpyrifos-degrading fungus *Cladosporium cladosporioides* strain Hu-01 (collection number: CCTCC M 20711), a fungal wettable powder preparation was developed aiming to efficiently remove chlorpyrifos residues from vegetables. The formula was determined to be 11.0% of carboxymethyl cellulose-Na, 9.0% of polyethylene glycol 6000, 5.0% of primary alcohol ethoxylate, 2.5% of glycine, 5.0% of fucose, 27.5% of kaolin and 40% of freeze dried fungi by response surface methodology (RSM). The results of quality inspection indicated that the fungal preparation could reach manufacturing standards. Finally, the degradation of chlorpyrifos by this fungal preparation was determined on pre-harvest cabbage. Compared to the controls without fungal preparation, the degradation of chlorpyrifos on cabbages, which was sprayed with the fungal preparation, was up to 91% after 7 d. These results suggested this freeze-dried fungal wettable powder may possess potential for biodegradation of chlorpyrifos residues on vegetables and provide a potential strategy for food and environment safety against pesticide residues.

## Introduction

Chlorpyrifos is a broad–spectrum, moderately toxic organophosphorus pesticide [1]. Since been first introduced into market in 1960s, chlorpyrifos has been globally used in pest control in agriculture and home for its acute neurotoxic effects through acetylcholinesterase inhibition and consequent cholinergic hyper stimulation [2], [3]. However, increasing evidences indicate that chronic exposure to chlorpyrifos can cause persisting neurobehavioural dysfunction [4], even with low doses, which can elicit chronic toxicity [5]. There is ample evidences that chlorpyrifos for controlling insects agriculturally or residentially can adversely affect non-target organisms like fish [6], bees [7], silkworms and rats [8]. These potential threats lead to a great concern of environment and food safety and human health for its potential toxic [9], [10]. Although the strict limitation of chlorpyrifos residue launched in many countries, the use of the pesticide is still extensive. Thus, it is urgent to develop an efficient and convenient strategy able to eliminate the chlorpyrifos residues on vegetables and soils so that the food and environment safety can be guaranteed.

Biodegradation, using living microorganisms or active enzymes to detoxify and degrade pollutants, has received attention as a safe, efficient and cost-effective approach to clean up contaminated environment [11]–[15]. The degradation of chlorpyrifos by microorganisms in pure cultures has been well investigated [16], [17] and all the published studies concluded that using active microorganisms could be a promising approach for chlorpyrifos degradation on farm products [16], [18]. However, field conditions adversely influence microbial ingredients and impair the degradation capacity, so little is known on chlorpyrifos degradation by microorganisms in agricultural application. How to apply these pesticide-degrading microorganisms in field conditions has always been the greatest obstacle. In fact, with the reported methods [19], like using cell-free extracts [18], it was still difficult to reach the expected performance and hard to put into field application.

The practical application of microorganisms with pesticide degrading capacity could refer to the microbial pesticides like *Bacillus thuringiensis* (BT), which have already been developed many commercial formulations including oil solutions, wettable powder and effervescent tablets [20]. Development of microbial preparations capable of degrading pesticides mostly depends on the persistence of active compounds, which could be improved by the formulation design and selection of assistant agents. However, the extraction of active proteins increases the manufacture cost and the producing procedure may impact the enzyme ability [21], thus a cost-efficient, effective and safe method of microbial preparation should be established.

In this study, using a chlorpyrifos degrading fungus *Cladosporium cladosporioides* strain Hu-01 (collection number: CCTCC M 20711), a fungal preparation able to eliminate chlorpyrifos residues was developed through a process of vacuum freeze dehydration with appropriate agents. In addition, the multiple factors could affect the preparation quality was evaluated and checked to ensure its being qualified for industrial manufacture and market promotion. The objectives of this study were to test and determine the capacity of the preparation to degrade chlorpyrifos residue on vegetables, and to explore a potential approach for chlorpyrifos degradation by microorganisms in field conditions.

## Materials and Methods

### Chemicals and fungus

Technical grade chlorpyrifos (96% purity) was obtained from Dow AgroSciences, USA. All other chemicals and solvents used were analytical grade.

Activated sludge samples were collected as inoculum from a pesticide-manufacturing wastewater treatment system and chlorpyrifos-degrading fungus *Cladosporium cladosporioides* Hu-01 was screened and isolated [16]. This strain was deposited in China Center for Type Culture Collection under the collection No. CCTCC M 20711. The constant-temperature culture method was referred to Gao et al. [22] and the conditions were inoculum amount 0.1% (wet fungi), 28°C and 150 rpm on a rotary shaker for 5 days. Previously, the degradation of chlorpyrifos by strain Hu-01 was determined. 92.7% of chlorpyrifos at the initial concentration of 25 mg·L^−1^ was degraded in half hour, which revealed that strain Hu-01 can significantly reduce chlorpyrifos residue.

### Chemical analysis

The analysis method of chlorpyrifos was referred to Gao et al. [22]. Chlorpyrifos was analyzed on an Agilent 1100 High Performance Liquid Chromatography (HPLC) (Agilent, USA) equipped with a Hypersil ODS2 C_18_ reversed phase column (4.6 nm × 250 mm, 5 mm). A mixture of methanol and water (90∶10, *v*/*v*) was used as the mobile phase at a flow rate of 1.0 mL·min^−1^. The injection volume was 10 µL.

Recoveries of 2.5, 5, 10, 25, and 50 mg·L^−1^ of chlorpyrifos were determined from 86.3% to 106.2% and relative standard deviation (RSD) ranged from 0.6% to 4.1%.

### Procedure of freeze-dried fungi powder

After culturing for 7 d, hyphae were collected by filtering. Being pre-freezed at −20°C for 24 h, the fungi samples were immediately vacuumed and freeze-dried for another 24 h. Then, those samples were kept in normal temperature and the preliminary preparation was done. The degradation of chlorpyrifos by dried powder was conducted by adding 0.1 g of fungi powder to 5 mL of 25 mg·L^−1^ chlorpyrifos in phosphate buffered solution and the residual concentration was determined in half hour by HPLC.

### Determination of protectants and carriers

To maintain the microbial activity, protectants were added before dehydration and each fungi-protectant sample was moved to freeze dehydration machine for 24 h until the humidity fell to 2% or 3%. The degradation of 25 mg·L^−1^ chlorpyrifos was then investigated by HPLC. Control groups without protectants were set and the HPLC results leaded to the available protective agents and the suitable dosages.

The biocompatibility of carriers including diatomite, kaolin, bentonite and white carbon were investigated by mixing 50 mL of 10% fungi solution with 500 mg·L^−1^ carriers, adjusting chlorpyrifos at 25 mg·L^−1^ and degradation rate of chlorpyrifos was determined after 24 h. Carriers were individually mixed with freeze-dried fungi (*v*: *v* = 2∶3) and then the status of blocking, fluidity and cost of each carrier-fungi powder sample was investigated by investigation and surveys. Control groups without carriers were set and the observation results leaded to the available carriers and the suitable dosages.

### Determination and composition optimization of dispersants and surfactants

The biocompatibility of fungi powder with assistant agents was tested including dispersants: gelatin, polyethylene glycol 6000 (PEG 6000), polyvinylpyrrolidone (PVP), carboxymethyl cellulose-Na (CMC-Na), CMC and Polyvinyl alcohol (PVA); and surfactants: primary alcohol ethoxylate (PAE), sodium dodecyl benzene sulfonate (SDBS), sodium dodecyl sulfate (SDS) and nonylphenol ethoxylate (NPEO). 50 mL of 10% fungi solution was mixed with 50 mg·L^−1^ dispersant or surfactant, adjusting chlorpyrifos at 25 mg·L^−1^ and degradation rate of chlorpyrifos was determined.

For the dispersants, the selection was carried out by pouring 5.000 g of each dispersant into 250 mL of standard hard water at 30°C ±2°C and letting it sit for 30 min after oscillation. Then move 9/10 of upper liquid out and hydrate the left 25 mL solution. Repeat 3 times. The weight of each sample was record and the dispersion rate was calculated by **Eq. (1)**.
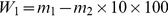
(1)where *W_1_* is dispersity (%), *m_1_* is the average mass of sample and *m_2_* is the average mass of hydrated sample of 25 mL left in bottom.

For the surfactants, wetting time of each one was determined. Pour 5.0 g of each surfactant-fungi powder sample into 100 mL of standard hard water. The duration it took to be fully wet was recorded and repeated 5 times. Then the average time was calculated and the available surfactants were determined.

Response surface methodology (RSM) based on the Central Composite Rotatable Design (CCRD) was applied to optimize the each key component as a variable [16]. Theoretically, the interactions among variables could significantly affect the dispersion of fungal powder preparation in solution and the degradation of chlorpyrifos can be influenced accordingly [23], [24]. In this experiment, three assistant agents for optimizing were PAE (3.0% to 7.0%), CMC-Na (5.0% to 13.0%) and PEG 6000 (5.0% to 13.0%). The range and center point values of three independent variables were based on the results of preceding experiments. The dependent variable was the dispersion rate determined by adding each 5.000 g agent via **Eq. (1)**. The data were analyzed using RSM of the statistic analysis system (SAS) software (Version 9.0) to fit the following quadratic polynomial equation (**Eq. (2)**).
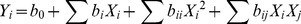
(2)where *Y*_i_ is the predicted response (dispersion (%) ), *X*_i_ and *X*_j_ are variables, *b_0_* is the constant, *b_i_* is the linear coefficient, *b_ij_* is the interaction coefficient, and *b_ii_* is the quadratic coefficient.

### Preparation of wettable freeze-dried fungi powder

The procedure of fungi preparation was carried out as following. Applied protectants were individually mixed with hyphae filtrated from fermented solution before freeze dehydration, and then the freeze-dried powder sample was mingled with other assistant agents. After altering pH to 6∼7 and smashing till 95% of it sieved to 63 µm, the final preparation was done and ready for degradation performance inspection.

### Preparation quality inspection

Protein concentration was measured by the method of Coomassie blue staining method with bovine serum albumin as a standard using a spectrophotometer (Shimadzu, Japan).

Wetting ability was determined by the time that 5.0 g of fungal powder took to be fully wet. Dispersion ability was inspected by the dispersion rate of fungal powder in solution. The size of powder granules was tested through the standard mesh of 325 (≥95%). Humidity was detected by weighing 0.5 g of fungal preparation and culture vessel together. After 2 h in dryer at 100°C, calculate the average humidity content using the weight of cooled preparation and culture vessel and repeated 3 times.

Storage stability was checked by storing for 15 d and 1 to 5 months at 4°C and 25°C, respectively, then reacting with chlorpyrifos at 25 mg·L^−1^ for 30 min and the degradation performance was detected to illustrate the stability of the preparation. The stability in low temperature was tested at 0°C in 1 h and 7 d, respectively.

### Degradation performance of freeze-dried fungal powder

Each test area of 4 m^2^ was divided from a cabbage filed without any pesticides applied before and 45% chlorpyrifos emulsifiable concentrate (EC) was sprayed at 225, 350, 450, 700 and 900 g·L^−1^, respectively, with the spray amount of 50 L·667 m^−2^. After 3 d, the fungal preparation was sprayed at the amount of 200 g·667 m^−2^ against the controls sprayed only water. The samples investigated were taken randomly in 1, 3 and 7 d. 25.0 g of each vegetable sample soaking in 50.0 mL of acetonitrile were decolored by 0.2 to 0.8 g of active carbon. After filtrating, partitioning with NaCl, clean-up, and concentration, residues dissolved in *n*-hexane was analyzed by Gas Chromatography (GC) HP-6890 with ECD and a capillary column of BD-1701, 30 m×0.32 mm×1.0µm. The temperatures of injection port, column and detector were 100°C, 220°C and 250°C, respectively. The flow of gas was as follows: nitrogen, 60 mL·min^−1^; air, 60 m L·min^−1^; hydrogen, 30 m L·min^−1^.

### Data analysis

All of the experiments were carried out in triplicate, and the results were the means of three replicates. Standard deviations were also determined using Statistic Analysis System (SAS). The significance (*p*<0.05) of differences was treated statistically by one-, two-, or three-way analysis of variance (ANOVA) and evaluated by post hoc comparison of means using lowest significant differences (LSD).

### Ethics Statement

No specific permits were required for the described field studies. No specific permissions were required for these locations. We confirm that the location is not privately-owned or protected in any way. We confirm that the field studies did not involve endangered or protected species.

## Results

### Chlorpyrifos degradation by freeze-dried fungi

After freeze dehydration, the degradation of 10% freeze dried fungi powder was determined and simply 40.9±0.3% of chlorpyrifos at the initial concentration of 25 mg·L^−1^ was degraded, which was significantly lower than the degradation rate of 92.5±0.1% before freeze dehydration.

### Protectants and carriers

To preserve the biodegradation ability of strain Hu-01, the available protective agents were selected. From the results in **Table 1**, it was shown that three protectants, fucose, glycin and glycerol, largely protected the freeze fungi from inactivation. With the increase of protectant content, the degradation of chlorpyrifos was gradually enhanced, which was significantly differed from the controls. 7.5% of glycerol revealed the best protective ability and nearly 80% of chlorpyrifos was degraded. However, the sample with glycerol was difficult to smash and dehydrate, so glycerol was not the available option. Fucose and glycine also presented as good protectants and more than half of chlorpyrifos was degraded when they reached the maximum testing amount. Concerning the factors above, 5.0% of fucose and 2.5% of glycine were selected to be the protective compounds in this fungal preparation for their protective performance, effective cost and a reasonable distribution of assisted agents.

**Table 1:** Effects of protectants on chlorpyrifos degradation by freeze-dried fungi.

Protectants	Amount	Degradation rate
	(%)	(%)
Fucose	2.5	44.9±5.3^i^
	5.0	50.1±6.6^g^
	7.5	60.2±5.2^d^
Glycine	2.5	48.7±1.6^h^
	5.0	52.5±1.8^f^
	7.5	56.5±6.3^e^
Glycerol	2.5	68.1±1.7^c^
	5.0	72.0±1.7^b^
	7.5	78.8±1.5^a^
CK	N/A	34.6±1.0^j^

Note: The data presented are means of three replicates with standard deviation, which is within 5% of the mean. Different letters indicate significant differences (*p*<0.05, LSD test). CK represents the control group without protectants.

**Figure 1:**
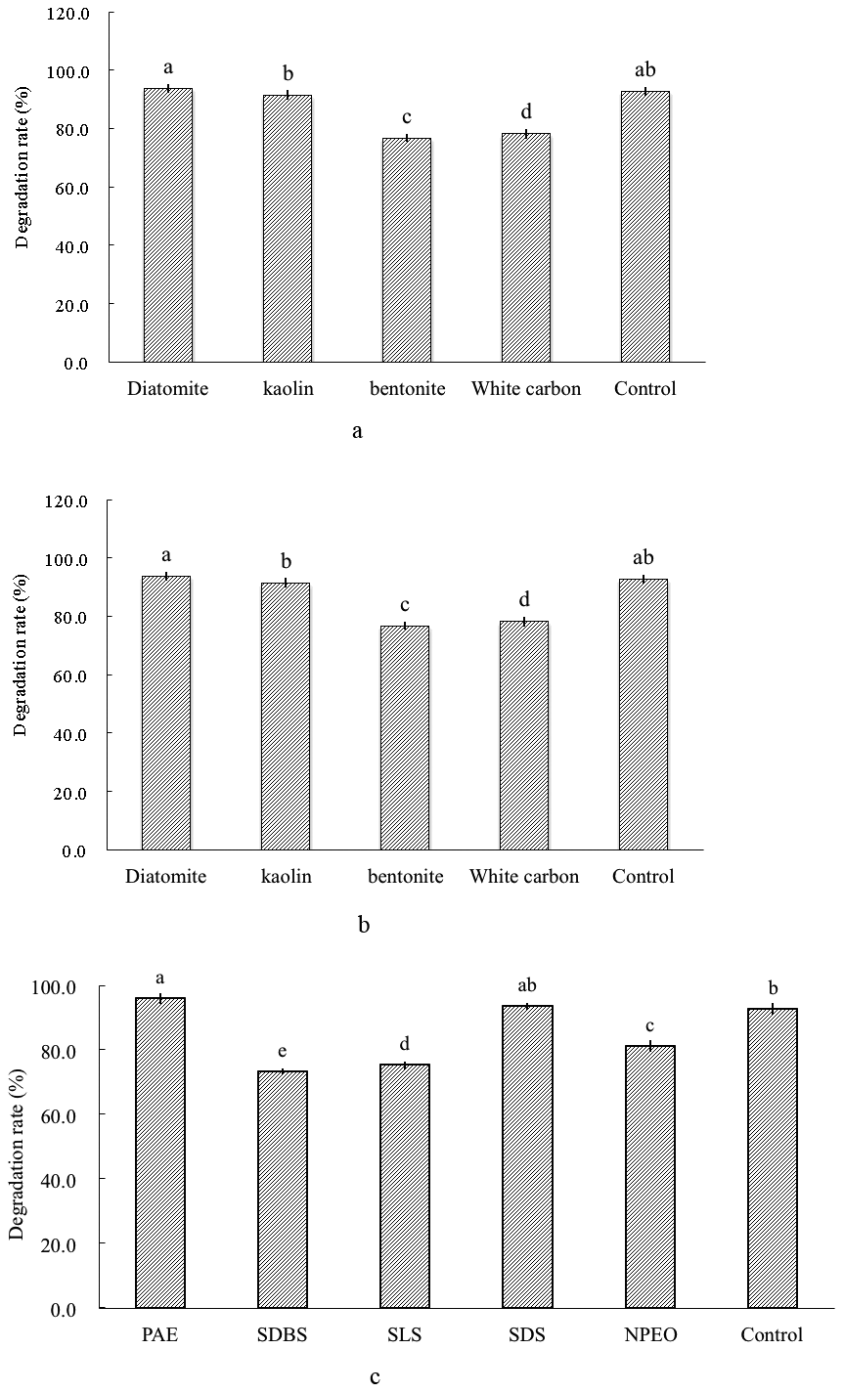
Effects of carriers, dispersants or surfactants on chlorpyrifos degradation added in freeze dried fungi. Note: a: effects of carriers; b: effects of dispersants; c: effects of surfactants.

The biocompatibility of tested carriers in **Fig. 1(a)** indicated that they were compatible with active fungi and no obvious disadvantages observed. From the observed results, the samples pre-treated by 27.5% of kaolin were easy to smash into powder with good fluidity while other three carriers were lack of fluidity or easily, even seriously blocking. As a result, kaolin was chosen to be the carrier in this process.

### Selection and composite optimization of dispersants and surfactants

The biocompatibility of dispersants and surfactants was investigated at first. The results in **Fig. 1(b and c)** illustrated that two categories of assistant agents were compatible with active fungi and no obvious disadvantages observed, which revealed that these agents in the present study contributed more or less to remain the degradation capacity of the strain and were suitable for further screening.

Then, four surfactants, SDS, SDBS, PAE and NPEO were evaluated. The time that each surfactant took to be fully wet was 63.7±2.8 s, 49.3±3.4 s, 10.6±0.7 s and 14.1±2.0 s, respectively, which indicated that PAE’s wetting period was shorter than the other three surfactants, so PAE was selected to be the surfactant in the fungal preparation.

Dispersants in the microbial preparation remarkably improve the thermodynamic stability. The results indicated that the samples with 5% of CMC-Na or PEG 6000 were the best in dispersion and ones with CMC, PAE and NPEO followed with obvious sediments or floccules. Hence, CMC-Na and PEG 6000 were recommended to be the available dispersants. The results from **Table 2** showed that 9% or 11% of CMC-Na or PEG 6000 revealed more excellent performance in dispersion with no significant differences.

**Table 2:** The dispersity of CMC-Na and PEG 6000 in different contents.

Dispersants	Content (%)	Dried fungi (%)	Dispersity(%)
CMC-Na	7	48	63.2±1.1^b^
	9	46	69.0±1.2^a^
	11	44	68.9±0.9^a^
PEG 6000	7	48	53.3±0.4^c^
	9	46	69.2±0.7^a^
	11	44	68.9±1.1^a^

Note: The data presented are means of three replicates with standard deviation, which is within 5% of the mean. Different letters indicate significant differences (*p*<0.05, LSD test).

Based on CCRD, RSM was employed to investigate the interactive effects of significant variables including CMC-Na (*X*_1_), PEG 6000 (*X*_2_) and PAE (*X*_3_) on dispersion performance. The design matrix and experimental responses for ingredient formulation are shown in **Table 3**. Subsequently, the data from **Table 3** were assessed by response surface regression procedure of SAS software package, and also the results of the quadratic polynomial model fitting in the term of ANOVA were shown in **Table 4**. By applying the multiple regression analysis on the experimental data, the following quadratic polynomial model equation (**Eq. (3)**) was delivered to explain the dispersion (*Y*) of dried fungal preparation (*p*>0.05):
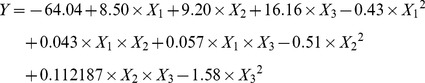
(3)where *Y* is the predicted dispersity (%) of the fungal preparation; *X_1_*, *X*_2_ and *X*_3_ are the coded values for the CMC-Na, PEG 6000 and PAE, respectively.

**Table 3:** Central composite rotatable design (CCRD) matrix and the response of dependent variable for dispersity of preparation (*Y*).

Run	Contents of variables (%)			Dispersity (%)
	*X*_1_	*X*_2_	*X*_3_	*Y*
1	11.0	11.0	6.0	61.42±0.84
2	11.0	7.0	4.0	61.13±0.66
3	7.0	11.0	4.0	64.09±1.25
4	7.0	7.0	6.0	65.09±0.58
5	11.0	11.0	4.0	60.57±1.84
6	11.0	7.0	6.0	62.49±0.48
7	7.0	11.0	6.0	65.62±1.10
8	7.0	7.0	4.0	64.37±0.55
9	11.0	11.0	4.0	56.87±1.62
10	11.0	7.0	6.0	63.11±1.14
11	7.0	11.0	6.0	65.04±1.04
12	7.0	7.0	4.0	65.82±1.68
13	11.0	11.0	6.0	61.87±1.22
14	11.0	7.0	4.0	61.23±1.73
15	7.0	11.0	4.0	64.20±0.98
16	7.0	7.0	6.0	68.67±1.05
17	9.0	9.0	5.0	65.92±2.97
18	9.0	9.0	5.0	69.83±0.91
19	9.0	9.0	5.0	68.58±0.79
20	9.0	9.0	5.0	71.04±0.85
21	5.0	9.0	5.0	59.98±0.69
22	13.0	9.0	5.0	72.07±2.02
23	9.0	5.0	5.0	59.05±1.93
24	9.0	13.0	5.0	70.41±0.54
25	9.0	9.0	3.0	60.68±17.99
26	9.0	9.0	7.0	72.66±2.09
27	9.0	9.0	5.0	72.04±0.27
25	9.0	9.0	3.0	60.68±17.99
26	9.0	9.0	7.0	72.66±2.09
27	9.0	9.0	5.0	72.04±0.27
28	9.0	9.0	5.0	71.83±0.40
29	9.0	9.0	5.0	72.60±0.49
30	9.0	9.0	5.0	72.51±0.63
31	9.0	9.0	5.0	72.46±0.67
32	9.0	9.0	5.0	71.60±0.85

Note: *X*_1_: CMC-Na; *X*_2_: PEG 6000; *X*_3_: PAE; *Y*: dispersity. The data presented are means of three replicates with standard deviation, which is within 5% of the mean.

**Table 4:** Analysis of variance (ANOVA) for the fitted quadratic polynomial model for dispersity of preparation.

Source	DF	SS	MS	*F* value	*Pr*>*F* *
Model	9	718.08	35.90	6.805	0.001
Linear	3	334.91	66.98	12.695	0.000
Square	3	342.93	68.59	12.999	0.000
Interaction	3	40.24	4.02	0.763	0.661
Error	13	58.04	5.28		
Total	22	776.12			

Note: DF refers to degrees of freedom; SS refers to sum of sequences; MS refers to mean square.

*P Level less than 0.05 indicate the model terms are significant.

The statistical significance of **Eq. (3)** was also evaluated by performing *F*-test and *t*-test (**Table 4** **and** **5**). The statistical analysis indicated that the model linear term coefficient of *X*_1_, *X*_2_ and *X*_3_ and the quadric term coefficient of *X*_1_, *X*_2_ and *X*_3_ showed significant effects (*p*<0.05) on dispersion. Thus, the quadratic polynomial equation (**Eq. (4)**) was modified to be:
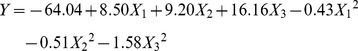
(4)


**Table 5:** Effect estimates for the fitted quadratic polynomial model for dispersity of preparation.

Term	Estimate	Standard Error	*t* value	*Pr>|t|* *
*X_1_*	2.64	0.47	5.633	0.000
*X_2_*	1.66	0.47	3.550	0.005
*X_3_*	1.83	0.47	3.909	0.002
*X_1_* * *X_1_*	−1.94	0.42	−4.566	0.001
*X_1_* * *X_2_*	0.17	0.57	0.301	0.769
*X_1_* * *X_3_*	0.12	0.57	0.201	0.844
*X_2_* * *X_2_*	−2.26	0.42	−5.330	0.000
*X_2_* * *X_3_*	0.22	0.57	0.391	0.703
*X_3_* * *X_3_*	−1.78	0.42	−4.186	0.002

Note: *X*_1_: CMC-Na; *X*_2_: PEG 6000; *X*_3_: PAE.

*P Level less than 0.05 indicate the model terms are significant.

**Figure 2:**
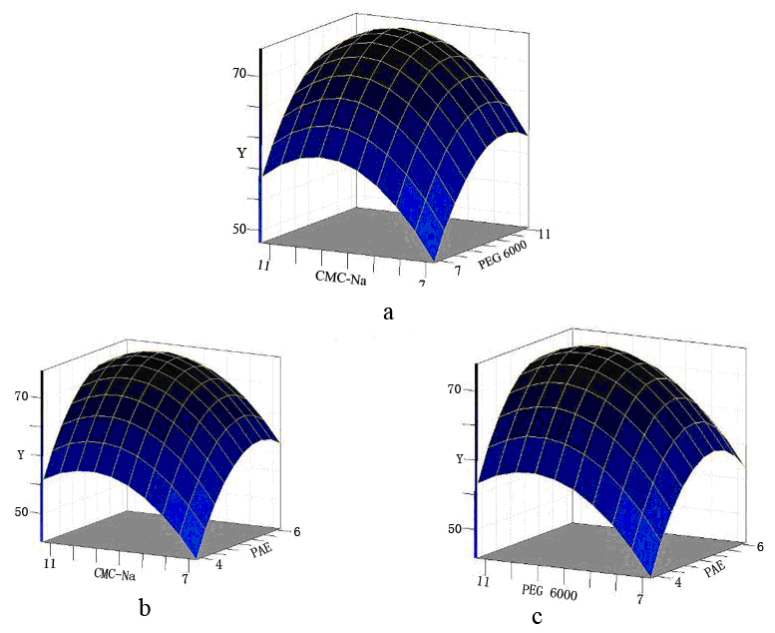
Response surface plots showing the effects of three variables on dispersity of the fungal preparation. Note: a: the effects of CMC-Na (*X*_1_) and PEG 6000 (*X*_2_) on dispersity (*Y*) of the preparation; b: the effects of CMC-Na (*X*_1_) and PAE (*X*_3_) on dispersity (*Y*) of the preparation; c: the effects of PEG 6000 (*X*_2_) and PAE (*X*_3_) on dispersity (*Y*) of the preparation.

The adequacy of two models was examined by the determination coefficients (*R* ^2^ = 0.9252), which suggested that the predicted values of the models were well correlated with the experimental values. The three-dimensional (3D) response surfaces in **Fig. 2** (**a**, **b** and **c**) show the respective effects of *X*_1_ and *X*_2_, *X*_1_ and *X*_3_, *X*_2_ and *X*_3_ on dispersion of fungal preparation while keeping the third variable at 0. The model predicted a maximum dispersion of 70.3% at the stationary point. Accordingly, the optimized formulation was determined by the predict model and previous experiment results to be 11.0% of CMC-Na, 9.0% of PEG 6000, 5.0% of PAE, 2.5% of glycine, 5.0% of fucose, 27.5% of kaolin and 40% of freeze dried fungi.

### Preparation quality inspection

To evaluate the quality of fungal wettable powder, the degradation of chlorpyrifos by optimized preparation was checked first and the results showed that it eliminated 76.1±0.4% of chlorpyrifos at the initial concentration of 25 mg/L in 30 minutes, which can meet the practical requirements. As the amount and concentration of total protein indirectly reflected the content of active protein, the equation (**Eq.** (**5**)) of the standard curve was obtained (*R* ^2^ = 0.9966) by Coomassie blue staining method:
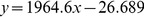
(5)where *y* is corresponding concentration of protein (mg·L^−1^), and *x* is the value of OD_595_. The value of *R* ^2^ suggested this model was stable and credible in the range of testing concentrations and the protein amount of freeze-dried fungi powder was 10.6 mg·g^−1^.

**Figure 3:**
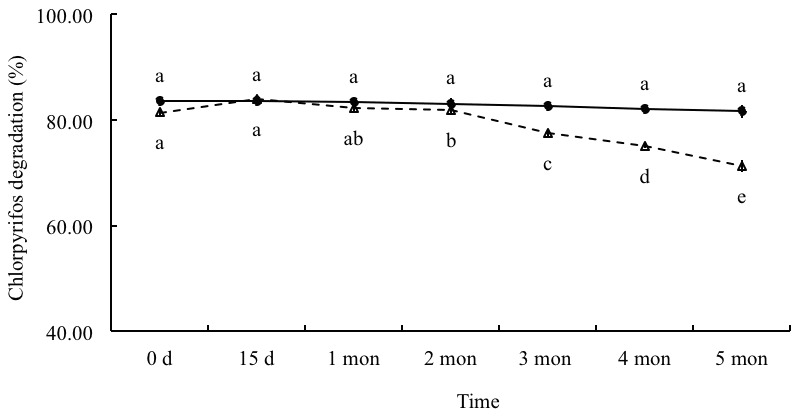
Chlorpyrifos degradation trends reflecting the stability of fungal preparation in normal and low temperature for different periods. Note: The data presented are means of three replicates with standard deviation, which is within 5% of the mean. Different letters indicate significant differences (*p*<0.01, LSD test). •, chlorpyrifos degradation by fungal preparation stored at 4°C; △, chlorpyrifos degradation by fungal preparation stored in normal temperature.

The results in **Table 6** indicated that low temperature (0°C) storage rarely affected the dispersing and degrading performances, which was non-significantly different (*p*>0.05) from ones stored in normal temperature. Meanwhile, stored at 4°C in periods of 15 d, 1, 2, 3, 4 and 5 months, the degrading performance was neither degenerated nor significantly different from ones before low temperature storage. Although after 5 months, the degradation rate dropped a little, the value remained over 70%, which could be expected to meet the practical needs (**Fig. 3**). Furthermore, other quality features of the chlorpyrifos-degrading preparation were investigated individually including wettability (<1 min), dispersion (77.8±1.6%), humidity (≤1.8±0.8%), fineness (≥95% of granules sieved through 63 µm) and pH value (6 to 7). All the results indicated that the fungal preparation was physically stable and available for practical use.

**Table 6:** The size, suspensibility and chlorpyrifos degradation of fungal preparation in normal and low temperature storage.

	Size(%)	Dispersity (%)	chlorpyrifos Degradation (%)
0 h	95.4±0.6^a^	77.8 n±1.6^b^	83.4±2.1^c^
0°C, 1 h	95.3±1.0^a^	74.7±1.4^b^	85.7±2.2^c^
0°C, 7 d	94.9±1.7^a^	74.6±1.3^b^	85.6±2.4^c^

Note: The data presented are means of three replicates with standard deviation, which is within 5% of the mean. Different letters indicate significant differences (*p*<0.05, LSD test). Size: the amount of preparation granules through standard sieve in 325 meshes.

### Chlorpyrifos degradation on cabbage by freeze-dried fungal powder preparation

The recoveries of chlorpyrifos on cabbage were obtained by adding chlorpyrifos at concentration of 0.01, 0.05 and 0.10 mg·kg^−1^ by GC and the results were 77.0%, 88.9% and 84.7%, respectively. As RSD ranged from 2.0% to 7.9%, the pre-treatment and detection methods of chlorpyrifos residue on cabbage were reliable and up the pesticide residue detection standards.

**Figure 4:**
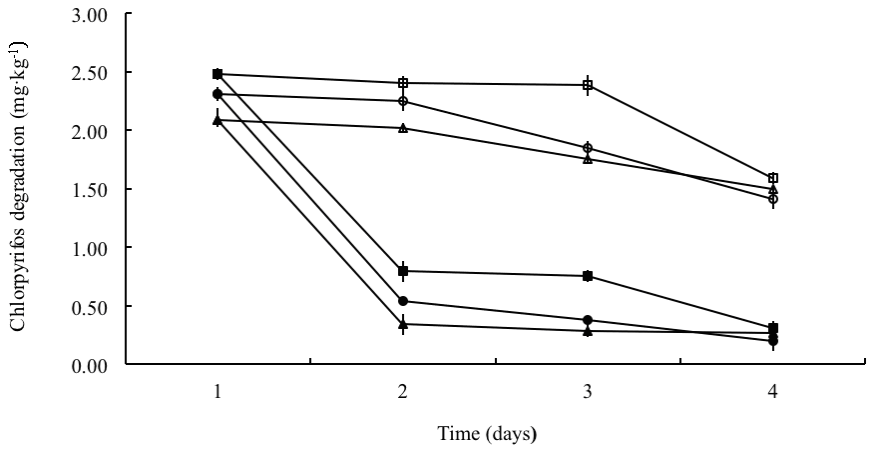
The trends of degradation of chlorpyrifos by fungal wettable powder on cabbage at different initial concentrations. The data presented are means of three replicates with standard deviation, which is within 5% of the mean: ▪, degradation of chlorpyrifos at initial concentration of 225 g·ha^−1^; •, degradation of chlorpyrifos at initial concentration of 350 g·ha^−1^; ▴, degradation of chlorpyrifos at initial concentration of 450 g·ha^−1^. Solid legends are treatments with fungal wettable powder while hollow ones are control groups.

From **Fig. 4**, the residual concentrations started at 2.12, 2.31 and 2.45 mg·kg^−1^, respectively in areas treated with 48% chlorpyrifos EC at the concentrations of 225, 350 and 450 g·ha^−1^. After applied with the microbial preparation in 1 d, the residual concentrations remarkably dropped to 0.31, 0.53 and 0.81 mg·kg^−1^, respectively while the residual concentrations of control groups were still over 2.0 mg·kg^−1^. In 3 d, residual concentrations decreased to 0.28, 0.37 and 0.76 mg·kg^−1^ while the control groups were 1.75, 1.85 and 2.38 mg·kg^−1^, respectively. In addition, the degradation rates grew up to nearly 87% within three days. In a period of 7 d, the chlorpyrifos residual concentrations constantly declined to 0.26, 0.21 and 0.29 mg·kg^−1^ while the control groups of 1.50, 1.41 and 1.59 mg·kg^−1^, respectively. Moreover, the degradation rates of each area increased to 87.3%, 91.0% and 88.2%, respectively.

## Discussion

Given the fact that biodegradation is an enzymatic reaction, the microbial activity of pesticide-degrading microorganisms is easily affected by external factors. Consequently, the technical difficulties of preparation design become the main obstacles of practical application and promotion. On the purpose of enhancing degradation activity and availability, the assistant agents, referred to the design of bio-pesticides, were introduced in this study. Assistant agents are all ingredients except the active compounds in microbial preparation and they help remain the extracted enzymes active and available [25], [26].

In this study, four categories of assistant agents including protectants, carriers, dispersants and surfactants were compared within group and the best options were determined from the results of four independent tests. Firstly, protectants preserved the fungi cells from cell damage caused by the process of freeze dehydration. The selection of available protectants was conducted with fucose, glycine and glycerol. From the test result, glycine and fucose were chosen out among applied agents. Glycine is a well-known protective compound for preventing tissue injury and enhancing anti-oxidative ability [26], [27] while fucose is safe in wound healing and protective against cytotoxicity [28], [29]. Secondly, carriers are the basic composites which are required to be high absorptive, fluid and cost-efficient [30]. By the test of physical features, kaolin was the best option for the carrier of this microbial preparation. Then, concerning the actual function of a final preparation, the wettability is vital [31]. This preparation was supposed to be sprayed on the surface of vegetable leaves and it was hard to soak the leaf efficiently because most plant blades are covered with wax which is the main reason of low surface energy on leaf surface. As a result, the surfactants were introduced. Common surfactants are anionic surfactants like ABS and PAE while some are nonionic surfactants like tween. Some natural products could be used as fine surfactants as well, for instance, lignosulfonates and saponin [32]. Through tests, PAE was selected for its shortest wetting time. Lastly, dispersants are regular assistant agents, which significantly decrease the gathering of granules in dispersion system. Generally, smashed preparations are not thermodynamically stable in water, which results in the automatical gathering of dispersed granules, called flocculation, to form a stable thermodynamic system [33]. However, the granules can be efficiently stopped from flocculating by dispersants which keep the active granules suspending in solution and remarkably improve the dispersibility and penetrability. Usually, dispersants are anionic surfactants with polycyclic structure, like lignosulfonates and alkylbenzene sulfonates (ABS). In the present study, CMC-Na and PEG 6000 were chosen out from tested dispersants because of their finest dispersity and compatibility in aqueous solution.

The strategy of response surface methodology (RSM) is commonly used in formula improvement and optimization [16], [34]–[36]. Since the function of fungal preparation is mostly relied on its property of dispersity, this strategy was applied for a better design of chosen surfactant (PAE) and dispersants (CMC-Na and PEG 6000). The mathematical model **(Eq. (4))** was obtained from the statistics analysis, which could be effectively used to predict and optimize the dispersity of fungal wettable powder preparation within chosen factors. Consequently, the optimum preparation formula was determined to be 11.0% of CMC-Na, 9.0% of PEG 6000, 5.0% of PAE, 2.5% of glycine, 5.0% of fucose, 27.5% of kaolin and 40% of freeze dried fungi. Under this formula, the dispersion rate was above 70.0% and approximately 76.1% of chlorpyrifos was degraded within half an hour in lab condition.

Assessment of preparation quality is necessary. Apparently, the impact of its physical and biological properties on the degradation performance of fungal preparation cannot be underestimated or ignored. Therefore, several features of product quality were checked separately and the results showed that the fungal preparation would meet the practical demands. However, the results still revealed an inevitable fact that microbial preparations are more possibly affected by external factors than the chemosynthetic ones. Moreover, this shortage was also evident in the field tests. Because of the vulnerability of active ingredients, a less efficient degradation performance of this fungal preparation was detected after one day, which can be considered as an indirect consequence of environmental factors on the active enzymes. As a result, these limitations will definitely enlarge the difficulty of its practical application and market promotion.

Undeniably, the results from the field tests showed a degradation of chlorpyrifos on cabbage more than 85% within one day, which indicated that the removal was efficient by this fungal preparation. In addition, it revealed the potential of degradation within a broad range of initial chlorpyrifos concentrations, which can be necessary because of the inappropriate and excessive use of chlorpyrifos in some farming areas in China. During the field experiments, there were no adverse effects observed on cabbage growth, which suggested that the fungal preparation was safe to the plants and can be an option for large-scale use in crops treated with chlorpyrifos. Lastly, as chlorpyrifos residues were constantly reduced, it was suggested that even though the efficiency was weakened after one day, this fungal preparation showed an important feature of persistently degrading the target compound.

In conclusion, the results obtained in the present study indicated that freeze-dried fungal wettable powder preparation of *C. cladosporioides* Hu-01 efficiently reduced the residues of chlorpyrifos on cabbage, which can be used as an effective and promising approach for chlorpyrifos bioremediation on vegetables. Besides, this study also provided a safe and cost-effective procedure for microbial preparation, which can be regarded as a potential strategy for better utilization of active microorganisms in bioremediation.
